# An epidemiological extension of the El Farol Bar problem

**DOI:** 10.3389/fdata.2025.1519369

**Published:** 2025-02-26

**Authors:** Francesco Bertolotti, Niccolò Kadera, Luca Pasquino, Luca Mari

**Affiliations:** School of Industrial Engineering, LIUC - Università Cattaneo, Castellanza, Italy

**Keywords:** agent-based modeling, El Farol Bar problem, social dilemma, epidemiological modeling, simulation, computational social science

## Abstract

This paper presents an epidemiological extension of the El Farol Bar problem, where both a social and an epidemiological dimension are present. In the model, individual agents making binary decisions—to visit a bar or stay home—amidst a non-fatal epidemic. The extension of the classic social dilemma is implemented as an agent-based model, and it is later explored by sampling the parameter space and observing the resulting behavior. The results of this analysis suggest that the infection could be contained by increasing the information available in the underlying social system and adjusting its structure.

## 1 Introduction

The appearance of COVID-19 pandemic brought a greater interest on the spread of epidemics in social systems, which has ascended as a research imperative (Squazzoni et al., 2020). The pandemic has especially highlighted the presence of an interplay between disease dynamics and socio-behavioral patterns (Kreulen et al., 2022). Consequently, understanding and strategizing against the spread of epidemics in interconnected social systems have become paramount to safeguarding global health and socio-economic stability (Maharaj and Kleczkowski, 2012).

Mathematical models (Kermack and McKendrick, 1927), and subsequently, simulation models (Bagni et al., 2002), have long been pivotal tools in epidemic management, offering the capacity to predict (Colizza et al., 2006), analyze (Colizza et al., 2007), and strategize (Ibarra-Vega, 2020) against the spread of infectious diseases (Ferreri et al., 2014). The computational implementation of an epidemiological model enables the analysis of disease transmission dynamics (Rahmandad and Sterman, 2008) through the systematic examination of epidemiological variables, even when they are not analytically tractable (Bobashev et al., 2007). In this perspective, simulations can serve two main interrelated goals, although a more precise taxonomy can be defined (Epstein, 2008; Edmonds et al., 2019). First, by incorporating real-world data and multifaceted parameters, simulations provide a computational platform to assess possible outcomes and interventions in real-world systems (Bertolotti and Roman, 2024). Second, simulations can be employed to assess the reliability of hypotheses and to refine the objectives of empirical studies and treatments (Georgescu, 2012).

The El Farol Bar problem (Arthur, 1994), a seminal example in complexity science (Casti, 1996), exemplifies the use of toy models to study the unpredictability of the dynamics of seemingly simple social systems (Lorenz, 1963). In the original form of the problem, multiple agents all face the same binary decision, that each of them has to make without the possibility to agree or to share information with the others: either to visit a bar with limited capacity or to stay home, where a threshold is set and known to all agents above which they no longer find it enjoyable to visit the bar. The binary outcome—either the visit was enjoyable if the bar was not too crowded or viceversa—is known to each attending agent after the event, and the time series of the outcomes of repeated events is the basis for predicting the next outcome and a decision of each accordingly.

In this paper, the dilemma has been modified and enrich with a new epidemiological dimension. Individuals must decide whether or not to engage in social activities amidst a contagious disease outbreak, as it happened during the COVID-19 pandemic (Kluwe-Schiavon et al., 2021). To the best of the authors' knowledge, interactions between the underlying mechanisms of social decision-making and the epidemiological dynamics in such scenarios are largely unexplored (Pullano et al., 2020), and the El Farol bar problem was never investigated from an epidemiological perspective.

The El Farol Bar problem extension has been implemented into an agent-based model (Bonabeau, 2002). This specific methodology embodies a bottom-up approach, allowing for the representation of heterogeneous behaviors and leading to the emergence of complex system-wide phenomena (Siegenfeld and Bar-Yam, 2020), and it is widely employed across multiple fields, including ecology (Goodman et al., 2023), economics (Arthur, 2006), social sciences (Marwal and Silva, 2023), and epidemiology (Squazzoni et al., 2020). The computational implementation was needed to generate simulated data, with the purpose of contributing to the understanding of the entangled nature of socio-epidemiological systems.

The results suggest that epidemic survival rate is strongly dependent on the experimental configuration, given that with the specific parameters' space employed only a small fraction of simulations resulting in persistent infections. This indicates that system dynamics are governed more by the setup of the configuration space than by external environmental factors. Parameters affecting infection survival display varied behaviors, monotonic and non-monotonic. Environmental co-effects highlight regions of increased infection persistence, particularly in configurations with higher population densities and prolonged infection durations. The results emphasizes the role of information flow and the structural dynamics of social systems, where short-term memory and agent presence significantly affect infection survival probabilities.

This paper is structured as follows: The agent-based model is first introduced and a detailed description of its components provided. The model exploration process is then outlined, emphasizing the methodology employed to generate the results. Finally, we present and discuss the outcomes and draw conclusions from our research.

## 2 Related works

Social dilemmas exist with the purpose of improving our understanding of how people interact in a resource-bounded environment (Van Lange et al., 2013), especially where there is a conflict between bounded rational entities which are metabolically dependent from a shared environment (Valentinov and Chatalova, 2016). At the best of our knowledge, Dawes (1980) was the first to introduce the concept of social dilemma, even thought a similar framework was already present in previous works (Hardin, 1968; Olson, 1974). Dawes describes social dilemmas as scenarios where individual decision-makers possess a dominant strategy that leads to non-cooperation and, if everyone adopt this dominant strategy, the outcome would be universally poorer, leading to a suboptimal equilibrium. The final rate of cooperation usually depends on the payoff structure (Rand et al., 2012); even then, it is an approximation and usually requires a certain number of iterations to be reached (Capraro, 2013).

The concept of the social dilemma is not confined to a single field of study. In economics, they encompass a range of perspectives and findings, such as investigating the difference between rational behavior and social norm (Weber et al., 2004; Biel and Thøgersen, 2007). Also, social norms have been employed for addressing environmental policies (Cerutti, 2017), conflict management strategies (Sitkin and Bies, 1993), social learning (Mobius and Rosenblat, 2014), and knowledge sharing (Razmerita et al., 2016). Ecology has a long tradition of using social dilemmas as well (Eshel and Motro, 1988; Woodall et al., 2000), especially in light of the pervasive presence of cooperation in natural species (Gokhale and Hauert, 2016). These settings can help in understanding the origin of sociality (Purcell et al., 2012) or group foraging strategies (Bach et al., 2006). Also, social dilemmas have been employed as the border between ecology and social sciences, to study how success in species conservation depends as much on individuals can collaborate to a common purpose (Di Chio et al., 2008; Cumming, 2018) and to use classic economic concepts such as signaling and contract theory to interpret evolutionary biology (Archetti et al., 2011).

While El Farol Bar problem (Arthur, 1994) is a classic example of social dilemma with decision-making under uncertainty, it already presents several extensions. Statistical approaches have been used to improve agents' decision-making strategies, particularly in dynamic environments with evolving population behaviors (Challet and Zhang, 1997). Also, many approaches have been proposed to model the prediction mechanisms and agent behaviors in contexts such as resource allocation (Challet and Zhang, 1997) and market dynamics (Johnson et al., 1998). A variant of the El Farol bar problem has recently been used to examine the fairness of social distancing measures introduced during the COVID-19 pandemic (Schosser, 2022). However, this analysis did not consider the potential for agents to become infected.

Although the fields of epidemiology and social dilemmas have not traditionally intersected extensively, recent years have seen a burgeoning interest in the interplay between these disciplines, particularly highlighted by global health crises such as the COVID-19 pandemic (Tanimoto and Tanimoto, 2018). The application of social dilemma frameworks has proven insightful for examining the relationship between individual behaviors and collective outcomes, notably in the context of vaccination rates within populations (Tanimoto, 2021). These analyses utilize various models to illuminate the impact of factors such as replicator dynamics (Kabir, 2021), social efficiency (Kabir, 2021; Khan and Tanimoto, 2023), and diffusion structures (Wei et al., 2019) on vaccination uptake. Human behavior has been indicated as critical in shaping epidemic trajectories (Ferguson et al., 2020), emphasizing how deviations from optimal behavior can lead to suboptimal public health outcomes (Meloni et al., 2011), how different cooperation styles affect the achievement of herd immunity (Gharakhanlou and Hooshangi, 2020), or the interplay between individual and collective behavior (Palomo-Briones et al., 2022). Furthermore, empirical studies highlight how cooperative behaviors may be amplified by the accelerated transmission of disease (Rychlowska et al., 2022). Additionally, the exploration of oscillatory behaviors within social dilemmas reveals how perceived infection risks can drive a collective shift toward more cautious approaches to social interaction, such as increased adherence to social distancing measures (Glaubitz and Fu, 2020).

## 3 Materials and methods

This section presents a description of the model of an epidemiological version of the El Farol Bar and the exploration methodology employed to generate results.

### 3.1 Agent-based model description

In this section an agent-based model of an epidemiological version of the El Farol Bar problem is described and the method employed to explore the model is presented.

In addressing how epidemics affects the social dynamics in the El Farol Bar problem, agent-based modeling serves for two compelling reasons: as an approach traditionally employed to address social dilemmas, it is an effective means of communication within the scientific community; and it is particularly well-suited for capturing individual behaviors and their effects on an overall epidemic spread. This enables to get insights from the global co-effect of individual (i.e., agent-related) epidemic and social variables.

**Figure 1:**
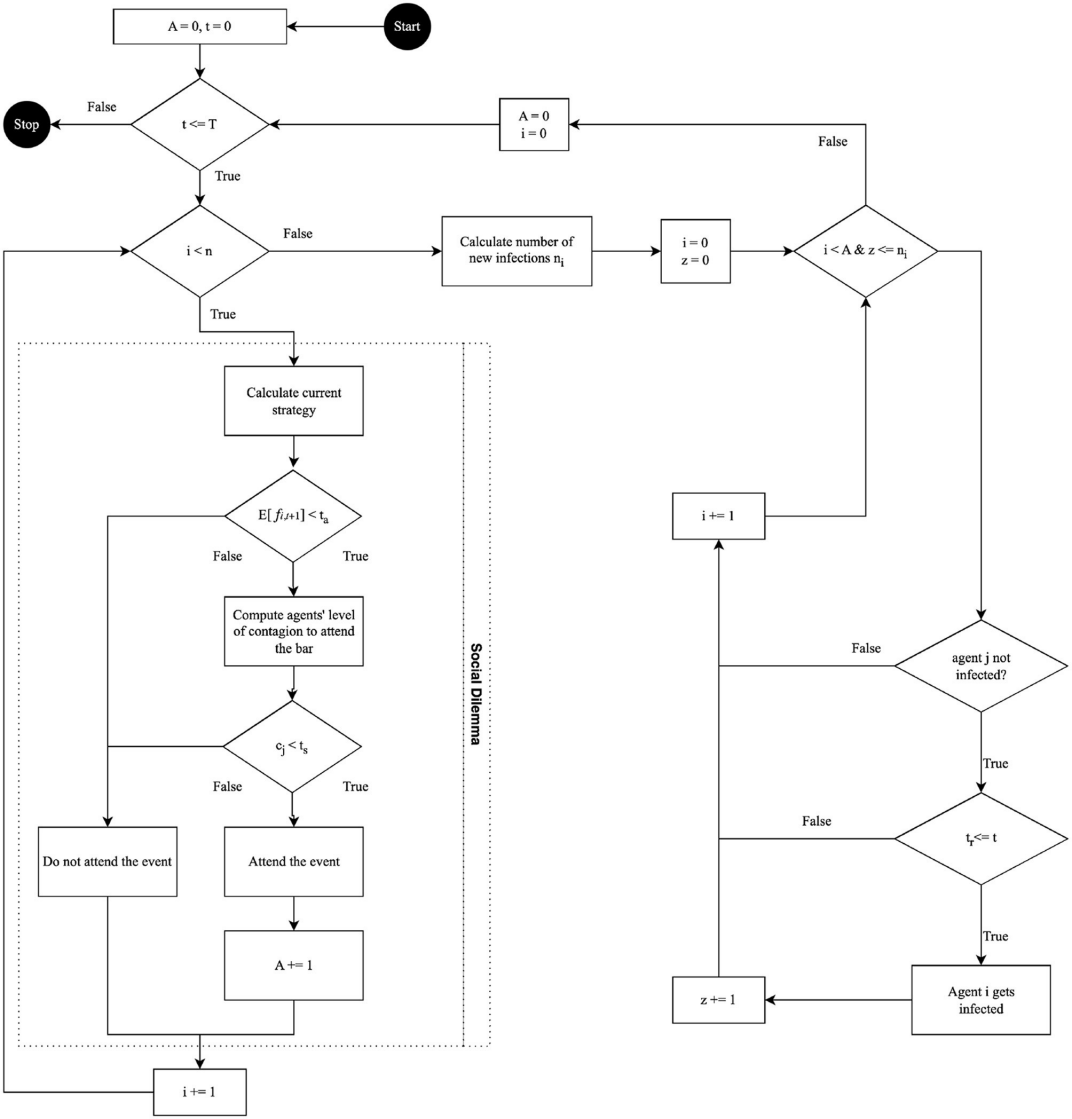
Scheduling process of the model.

At each time step *t*, the model implements a sequence of actions, as depicted in the flowchart (see Figure 1). The first concerns the decision-making process regarding bar attendance: evaluating agents' memory of past attendance, estimating the expected crowd level, and making a decision accordingly. The second is about the dynamics of infection as induced by the interactions among the agents given their health states, where an infectious pathogen could be transmitted to those who decide to attend the bar, influenced by the density of the crowd and the duration of exposure.

Together, these two sets of actions capture a dual aspect of agent behavior: social decision making influenced by past experiences and the epidemiological implications of these social choices. The model thus provides a framework for examining the interplay between individual decision making based on memory of previous states and the collective outcomes in terms of disease transmission, offering insights into how individual behaviors aggregate to impact public health.

#### 3.1.1 Social dilemma

The proposed model includes a single kind of agents, representing the individuals that could decide to attend the bar in any given week (the time step of the model) and thus possibly be infectious. The agents' behavior is modeled according to the hypotheses of the original El Farol Bar problem. First, the only decision each agent can make each week is whether to attend the bar, and the decision is always executed. Second, agents like to attend the bar, if it is not too crowded, and do it as much as they can: hence, each agent decides each week whether to attend the bar depending upon its expectation of the total number of agents who will attend. Third, agents interact with each other only at the bar, and therefore when their decision to attend has been already made.

The model introduced by this paper incorporates several hypotheses concerning agents' behavior. First, agents make a binary decision, as they can either choose to attend a bar or not; no other actions are included in the model, to focus on a specific aspect of social behavior. Second, agents inherently enjoy attending the bar and will do so as much as they can, but their preference is tempered by the bar's occupancy; agents are averse to overcrowding. Therefore, the decision to attend the event is influenced by their expectations regarding how crowded the bar will be. In time, this introduces a feedback loop where the average attendance of the bar inversely affects its attractiveness while it is directly influenced by it; a dynamic seen in many real-world social scenarios. Third, agents' interaction is solely defined by the shared presence in the crowded space of the bar, and there are no interpersonal communications or relationships affecting their attending decision.

The information about past attendance plays a crucial role in shaping agents' expectations, as it is used to estimate the number of agents likely to attend the bar in the subsequent week, as follows. For agents attending the event, the new value is the actual number of agents at the bar, while for agents that did not attend the new element of the memory is a random value, which stands for an educated guess made by agents which can not communicate with each other.

The agent's decision whether to attend the event or not is taken comparing an attendance threshold and the expectation regarding the future attendance. The attendance threshold *t*_*a*_ is a parameter of the model that depicts venue saturation level above which agents would consider unpleasant to be in the bar, consequently not attending the event.

Each agent *i* (where *i* goes from 1 to *n*, the total number of agents) generates an expectation regarding how many agents will attend the bar at the next time step memorizing the number of agents present at the bar during the last *m* times it attended the bar, with *m* being the memory length, and assigning weights to generate a prediction. In cases where the agent does not attend the bar, the value saved in memory is the one hypothesized by the agent, namely, the one generated with the 'expected attendance'. Let *s*_*i, k*_ be the *k*-th element of the memory of agent *i* (with *k*∈{0, 1, ..., *t*}) and *w* the list of weight *w*_*k*_ used to the define importance of each memory element, which increases with *k*. So, the attendance—which is the number the agent *i* expects to be at the event at time *t*+1—is therefore given by:


(1) $$\begin{array}{l} E_{i}\left[\overset{n}{\underset{j=1}{\sum }}a_{j,t+1}\right]=\overset{m}{\underset{k=1}{\sum }}s_{i,k}w_{k} \end{array}$$

where *a*_*j*_ is the participation of the agent *j* to the event at time *t*+1, $\overset{n}{\underset{j=1}{\sum }}a_{j,t+1}$ is the total attendance at time *t*+1 and $E_{i}\left[\overset{n}{\underset{j=1}{\sum }}a_{j,t+1}\right]$ is the expectation of the total attendance from agent *i* at time *t*+1. Consequently, from the expected attendance is possible to determine also the expected filling *f* of the venue at the time *t*+1


(2) $$\begin{array}{l} E_{i}\left[f_{t+1}\right]=\frac{E_{i}\left[\sum_{j=1}^{n}a_{j,t+1}\right]}{C_{max}} \end{array}$$

where *C*_*max*_ is the maximum capacity of the venue. Given the expected filling, at each time step an agent *i* attends the event whenever *E*_*i*_[*f*_*i, t*+1_] < *t*_*a*_.

#### 3.1.2 Epidemiological transmission

**Figure 2:**
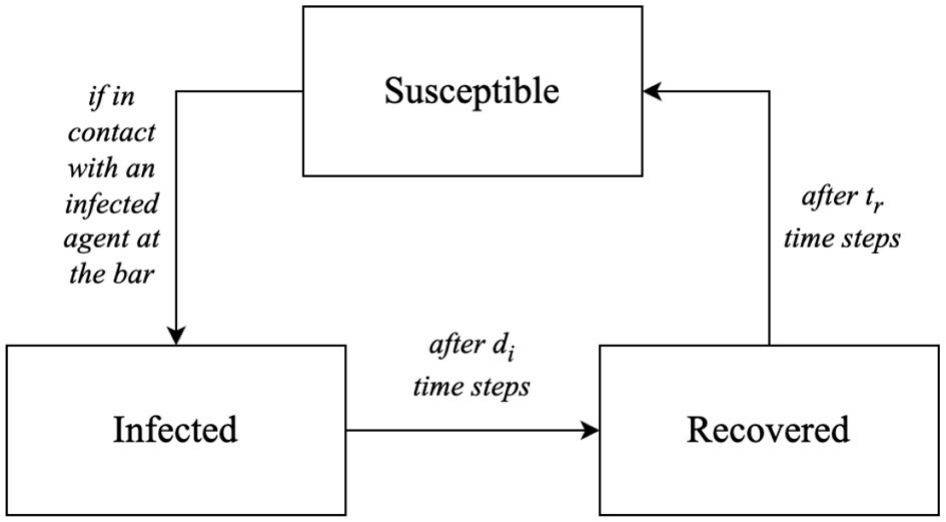
Map of possible infection status and their transitions.

In epidemiological models, each agent is typically (but not necessarily) in one of three states: susceptible, infectious, or recovered. These class of models accounts for the possibility of waning immunity after an infection, and eventually become susceptible again, modeling diseases where immunity, either natural or vaccine-induced, can be acquired and diminishes over time. Figure 2 depicts this transition.

The epidemiological dimension of this model is based on several key modeling hypotheses. Firstly, the contagion process is assumed to be uniform across all agents, characterized by a consistent duration and an initially uniform level of infectiousness. This simplification negates individual variations in disease progression and response to infection. Secondly, the model posits that the disease in question is non-lethal; agents cannot die as a result of contracting the illness. This assumption is critical as it focuses the model on the dynamics of disease spread rather than mortality rates, and the overall number of individual in the system remains the same. Despite the absence of mortality and the maintenance of agent numbers over time, a disease that infects multiple agents has the potential to reduce the number of agents available for social interaction at any given time. Furthermore, the model assumes the absence of long-term physical or psychological effects post-infection. Recovered agents are not hindered in their ability to participate in normal activities, such as attending a bar, indicating that the disease does not cause lasting health impacts. Psychologically, the model assumes that agents do not experience fear or behavioral changes as a result of the infection. They continue to frequent the events without any alteration in their behavior due to the experience of being infected. The sole variable that the agent takes into account when determining whether or not to attend the bar is their own contagion level *c*_*i*_. Indeed, should an agent's *c*_*i*_ be too high, they may elect not to attend the bar due to the symptoms of the disease. Finally, a crucial aspect of this model is the agents' ignorance of the epidemic. Agents lack information about the total number of infected individuals and do not consider the risk of infection in their decision-making process. This implies a lack of adaptive behavior in response to the epidemic, which significantly influences the model's predictions about disease spread. By ignoring potential changes in social behavior and risk assessment, the model strictly focuses on the deterministic spread of the disease under constant behavioral patterns. This approach simplifies the modeling process but may overlook important dynamics present in real-world scenarios where awareness and behavioral adaptations play a crucial role in disease transmission.

Agents can get infected only by participating in an event. So, the epidemic transmission happens solely at the bar, and only if at least one infectious is attending. The number of new infected agents *i*_*t*_ at time *t* is


$$\begin{array}{l} i_{t}\propto \left\lfloor \alpha \frac{\sum_{j=1}^{n}a_{j}c_{j}S_{t}}{C_{max}}\right\rfloor \end{array}$$

where is *S*_*t*_ the number of agents in susceptible state attending the bar at time *t*, and α is the level of contagiousness of the infection.

In the proposed model, social relationships among agents are not considered, leading to a uniform infection probability for all individuals attending the bar at time *t*. Consequently, the selection of new *i*_*t*_ infected agents at each time step is randomized from those present, not considering individual interactions or relationships.

Whenever, an agent becomes infected, the infection follows this dynamics. Initially, the contagion level of the newly infected agent *i* is set to *c*_*i*_ = 1. Given an infection duration *d*_*i*_, the contagiousity of agents decreases linearly by 1/*d*_*i*_ at each time step.

**Figure 3:**
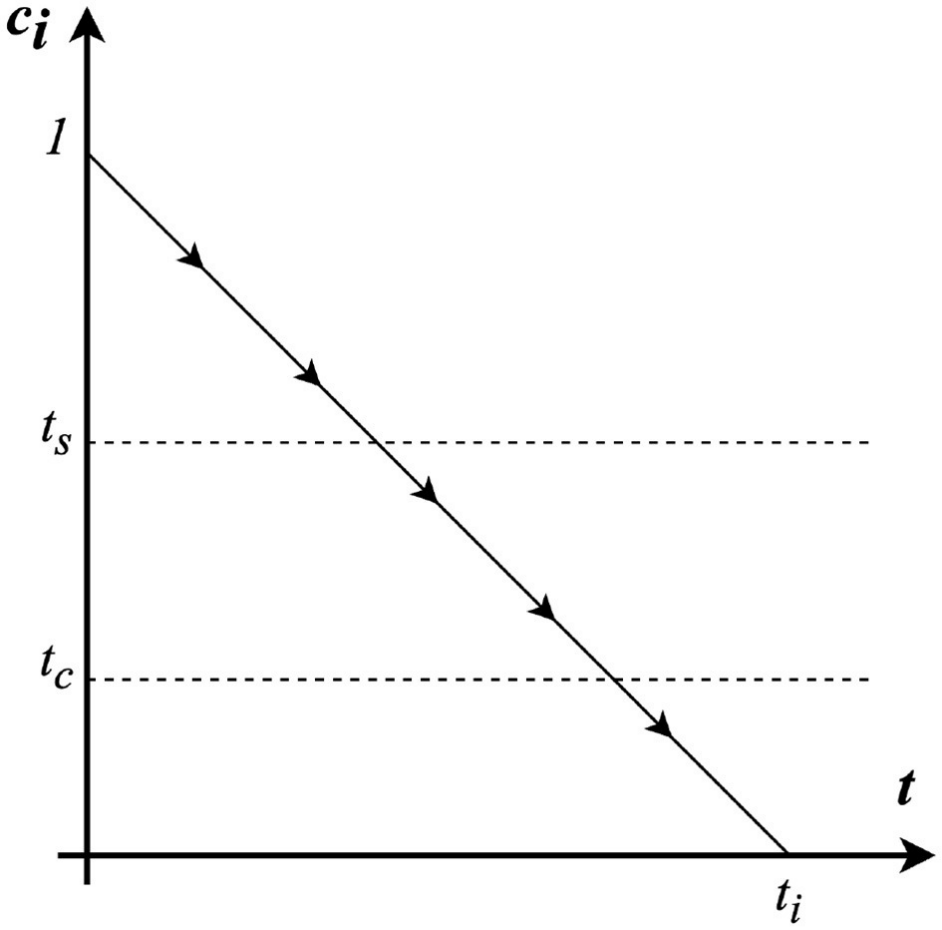
Dynamics of contagious level for each agent.

In the progression of the disease modeled, two critical thresholds, *t*_*s*_ and *t*_*c*_, play pivotal roles in influencing agent behavior and the spread of the infection, as outlined in Figure 3. The first threshold, *t*_*s*_, represents the infection level at which an agent exhibits sufficient symptoms to deter them from attending the bar. The second, *t*_*c*_, indicates the infection level beyond which agents can spread the infection. The spread of the infection is most influenced by the agents with *t*_*c*_<*c*_*i*_<*t*_*s*_, so with a contagious level between these two thresholds. This is because it encapsulates the period when agents are infectious but may not have anymore the level of symptom severity or self-awareness to avoid social gatherings, thereby contributing to the disease transmission dynamics. Notable, the contagious level is taken into account only for the *n*_*i*_ agents which infection level is greater than *t*_*c*_.

In the modeled scenario, infected agents undergo a recovery process after a duration of *t*_*i*_ time steps. Upon recovery, these agents are conferred a temporary immunity lasting *t*_*r*_ time steps. However, this immunity is not permanent; after the elapse of *t*_*r*_ time steps, the agents once again become susceptible to infection. In this model, the transition from immune to susceptible is unambiguous; thus, an immune agent will invariably possess a null probability of becoming infected. This cyclical pattern of recovery and renewed susceptibility underscores the transient nature of immunity within the model.

### 3.2 Model exploration settings

The results from the simulation model were generated by means of a grid sampling exploration of the parameter space, to collect the model outputs from different parameters' combination (Collins et al., 2013). Grid sampling from a parameter space involves systematically selecting a finite subset of parameter values that aims at comprehensively represent the entire parameter space. The idea underlying the use of this technique is to facilitate the exploration of system behavior across distinct parameter combinations, especially in cases where not a specific behavior is expected or researched. Table 1 presents the parameters tested in the simulation and their explored ranges. Each parameter has been sampled from a uniform distribution. The data are collected by simulating the agent-based model 100, 000 times. A preliminary analysis was conducted to assess the sensitivity to seed variation with specific parameter combinations (Bertolotti et al., 2020). It was observed that the variability of the metrics due to seed changes was practically zero, indicating that the mean or the presence of interesting behavior in the time series was not dependent on the random number generator. Consequently, multiple experiments were not conducted for each random seed.

**Table 1:** Description of model parameters.

**Parameter**	**Description**
*t*_*a*_	Threshold of share of expected agents above which an agent does not attend the event
*d*_*i*_	Duration of the infection
*t*_*p*_	Threshold of infection above which the infected agents have symptoms and do not attend the bar
α	Level of contagiousness of the disease
*t*_*c*_	Threshold of infection below which an agent can not transmit the disease anymore
*m*	Weight of the last memory in the decision-making process of agents
*t*_*r*_	Duration of the immunity
*n*	Number of agents in the simulation

Even if the model is stochastic, each simulation was initialized with a specific random seed, that was stored as well. Consequently, the results were reproducible at a later stage, and the time series for each configuration of interest was observable. Furthermore, the initial number of infected is not fixed; rather, it is 10% of the population. This ensures that the infection will commence, and that any subsequent extinction will not be contingent on the initial number being insufficient, even if preliminary analysis has shown that this value does not have an effect on the results.

From each simulation run, two time-series were collected: *A*, which is the number of attending agent at each time-step *t*, and *I*, the number of infected agents at each step of the simulation. From each time-series two output were extracted: the mean value of the series *E*[*A*] and *E*[*I*], which are used to assess the overall status of the system in time.

The model, the exploration code and the data analysis are all implemented in Python 3.11.

## 4 Experimental results

**Figure 4:**
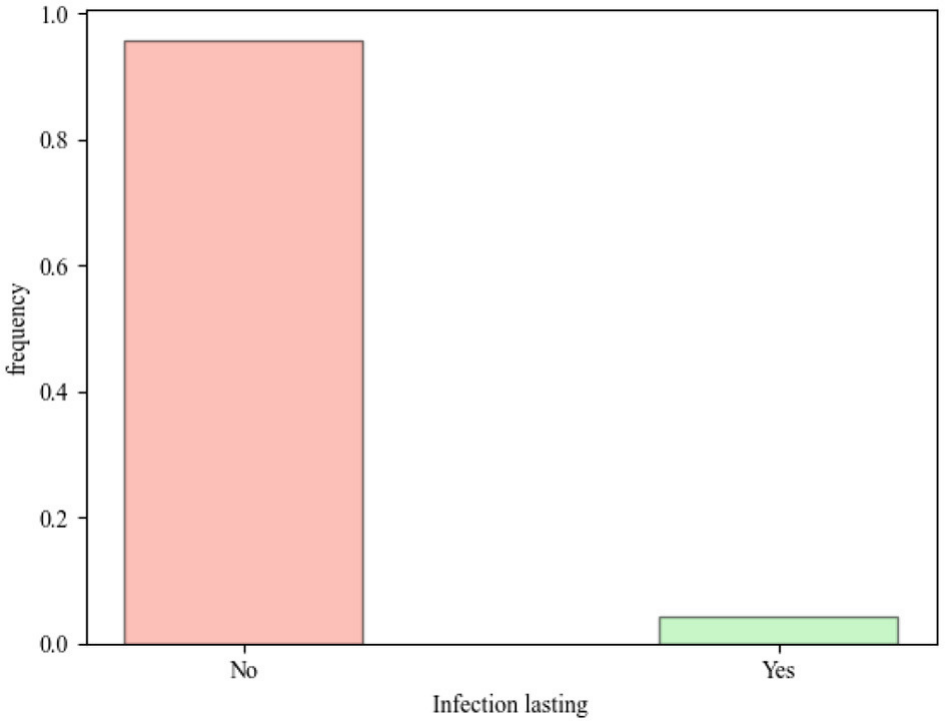
Share of runs where *I*(*t* = 200) > 0, i.e. where the infection lasted until the end of the simulation.

As illustrated in Figure 4, the epidemic did not persist until the conclusion of the simulation in the majority of instances when the specific experimental configuration was employed. The specific configuration of the model exploration plays a more significant role than the specific features of the model or the specific environmental parameters in determining the outcome. The percentage of surviving infections is highly dependent on the experimental configuration. Although it could be adjusted to achieve higher survival rates, the primary objective was to study the behavior of the system under the current conditions. Keeping specific configurations that increase the likelihood of infection survival would limit the study of dynamics and introduce bias by artificially inflating survival rates.

**Figure 5:**
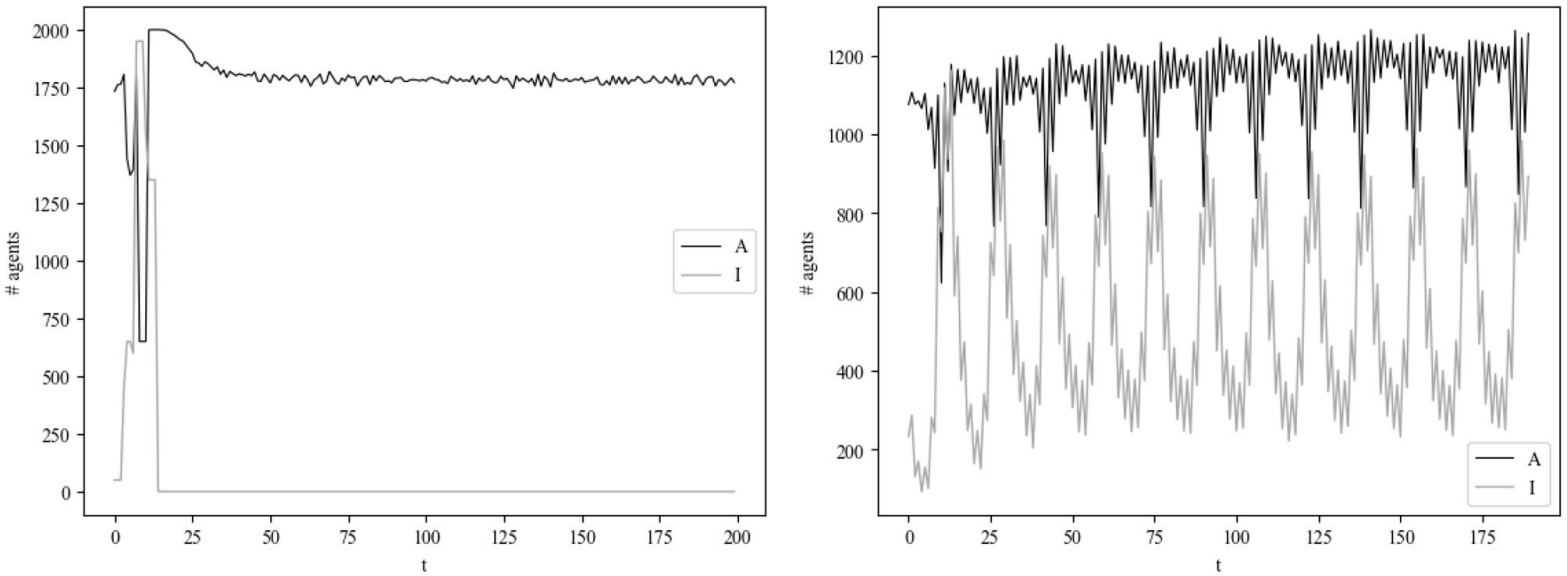
Behavior of *A* and *I* in two four different simulations. In the simulation on the left, the infection does not last to the end, which is what happens in the simulation on the right.

One might be surprised that only a small proportion of runs result in a lasting infection (Figure 5 shows an example of the model time series for a case where the infection lasted until the end and where it did not), and thus conclude that the existence of infection in social systems is merely coincidental. This line of reasoning fails to consider the potential for biological specimens to adapt, which could result in the emergence of new infectious diseases. Such adaptations may occur through evolutionary processes, resulting in the evolution of new pathogens that are better equipped to survive and disseminate within a given environment. An alternative hypothesis is that these adaptations could either result in the evolution of a more resilient pathogen or in the extinction of the pathogen. In a scenario where the infecting entity is not adapting, the observed consequences are the result of a previous adaptation. Therefore, the parameters configuration in Table 2 should be considered in this context, particularly with regard to the epidemiological elements.

**Table 2:** The following table presents the results of the simulation.

**Parameter**	***E*[*par*|*S*_*a*_]**	***E*[*par*|*S*_*i*_]**	** $\frac{E\left[par|S_{i}\right]-E\left[par|S_{a}\right]}{E\left[par|S_{a}\right]}$ **
*t*_*a*_	0.50	0.58	0.17
*t*_*c*_	0.50	0.17	−0.66
*d*_*i*_	5.51	6.91	0.26
*m*	0.50	0.39	−0.22
*t*_*p*_	0.50	0.56	0.13
*t*_*r*_	5.01	4.20	−0.16
α	0.26	0.32	0.23
*c*_*p*_	0.75	0.77	0.02
*n*	2252.35	2704.81	0.20

The second and third columns indicate the mean value of each analyzed parameter for the *S*_*a*_ and *S*_*i*_ scenario, respectively. The final column shows the relative difference between the two scenarios, with the values normalized by the base scenario.

**Figure 6:**
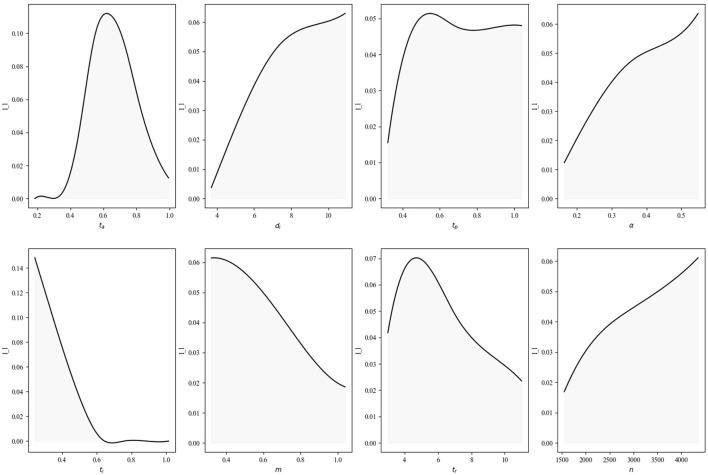
Distribution of experimental parameters for *S*_*i*_.

It is then possible to label of the simulation outcomes into two distinct groups based on their characteristics and behaviors;: *S*_*a*_, which includes all the simulation results, serving as a comprehensive dataset to which to make confrontations; and *S*_*i*_, containing those simulations where the epidemiological output *I*(*t* = 200) > 0. Table 2 provides a detailed comparison between two scenarios, and it was constructed by collecting the mean values of the input parameters, segmented into the two observed output scenarios. Given that these mean values were generated from a random grid sampling experiment across the parameter space, the difference can identify parameters' effect on scenario output. The third column of the table represents the relative difference between the mean input values for the two scenarios, providing a quantitative measure of each parameter's influence on the system's behavior. These quantitative results are also reflected in the description of Figure 6, where they are discussed later.

Figure 6 shows the effect of each parameter to the infection surviving, illustrating the distribution of each parameter exclusively for simulation runs for *S*_*i*_. Given that each parameter has been sampled from a uniform distribution, the presence of diverse shapes suggests that there is an actual effect of each parameters upon the infection continuation, and that this effect may vary. It confirms the finding from Table 2.

For instance, the infection duration (*d*_*i*_), contagiousness of the infection (α), and the number of agents in the simulation (*n*) all exert a growing monotonic influence on the infection's ability to survive. While the effect of *d*_*i*_ and α is intuitive, the impact of an increasing number of individuals is less clear, especially given that the capacity is not fixed but depends on *n*. This observation could depend on increased opportunities for interactions between agents, the potential for delayed saturation, and the presence of a buffer for randomness. Conversely, the significance of the most recently encoded event in the decision-making process of the agents (*m*) is univocally monotonically decreasing, indicating that a more rapid reaction time could diminish the probability of an infection's survival, even when considering solely the overall attendance and the absence of knowledge regarding the number of infected agents.

The remaining four parameters exhibit non-monotonic behavior. The threshold of contagion below which the infection is not spread (*t*_*c*_) or not perceived (*t*_*p*_) plays a role only for specific values, functioning as enabling factors. Finally, it can be observed that the attendance threshold, *t*_*a*_, and the recovery time, *t*_*r*_, exhibit a non-symmetric "bell" shape. In the first case, the dependence may be attributed to the fact that insufficient attendance does not result in the establishment of a critical mass at the bar, thereby preventing the constant dissemination of the epidemic. Conversely, when the value is excessively high, the epidemic rapidly eliminates all susceptible individuals, leading to extinction following a primary surge. With regard to the recovery time, it is evident that the proportion of surviving infections declines as this variable increases. However, it is counterintuitive that, at low values of this variable, there is an increase in the proportion of surviving infections. It may be the case that this is due to the fact that it reduces the size of the infection waves by reducing infection synchronization, thus allowing them to persist for longer without being extinguished due to the absence of non-infected agents.

**Figure 7:**
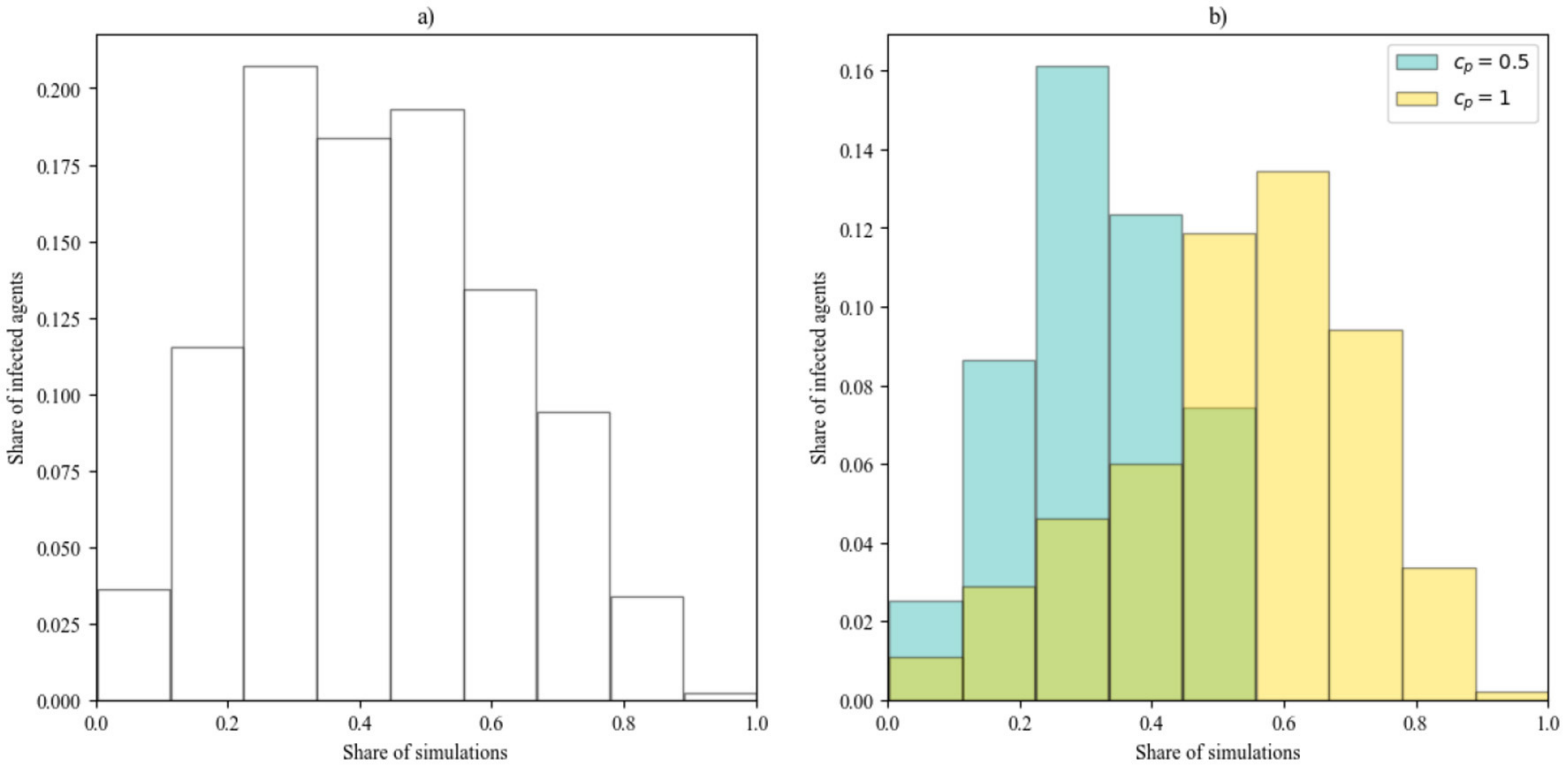
The distribution of the share of infected agents at the conclusion of the simulation *I*(*t* = 200) for *S*_*i*_, aggregated **(A)** and divided by the percentage capacity of the venue relative to the total number of agents *c*_*p*_ **(B)**.

In Figure 7, histogram on the left depicts the mean number of agents infected over the course of a simulation in which the infection was sustained, while the one on the right decomposes the data as a function of the percentage capacity of the bar, which can be only *c*_*p*_ = 0.5 or *c*_*p*_ = 1. The symmetrical shape of the histogram (a) could suggest a relatively balanced distribution of infection rates, with few extreme cases of very low or very high infection levels, but histogram (b) reveals that it depends strongly from the bar capacity, given that it is the combination of two distribution ranging from 0 to 1 with respectively a left and a right tail.

**Figure 8:**
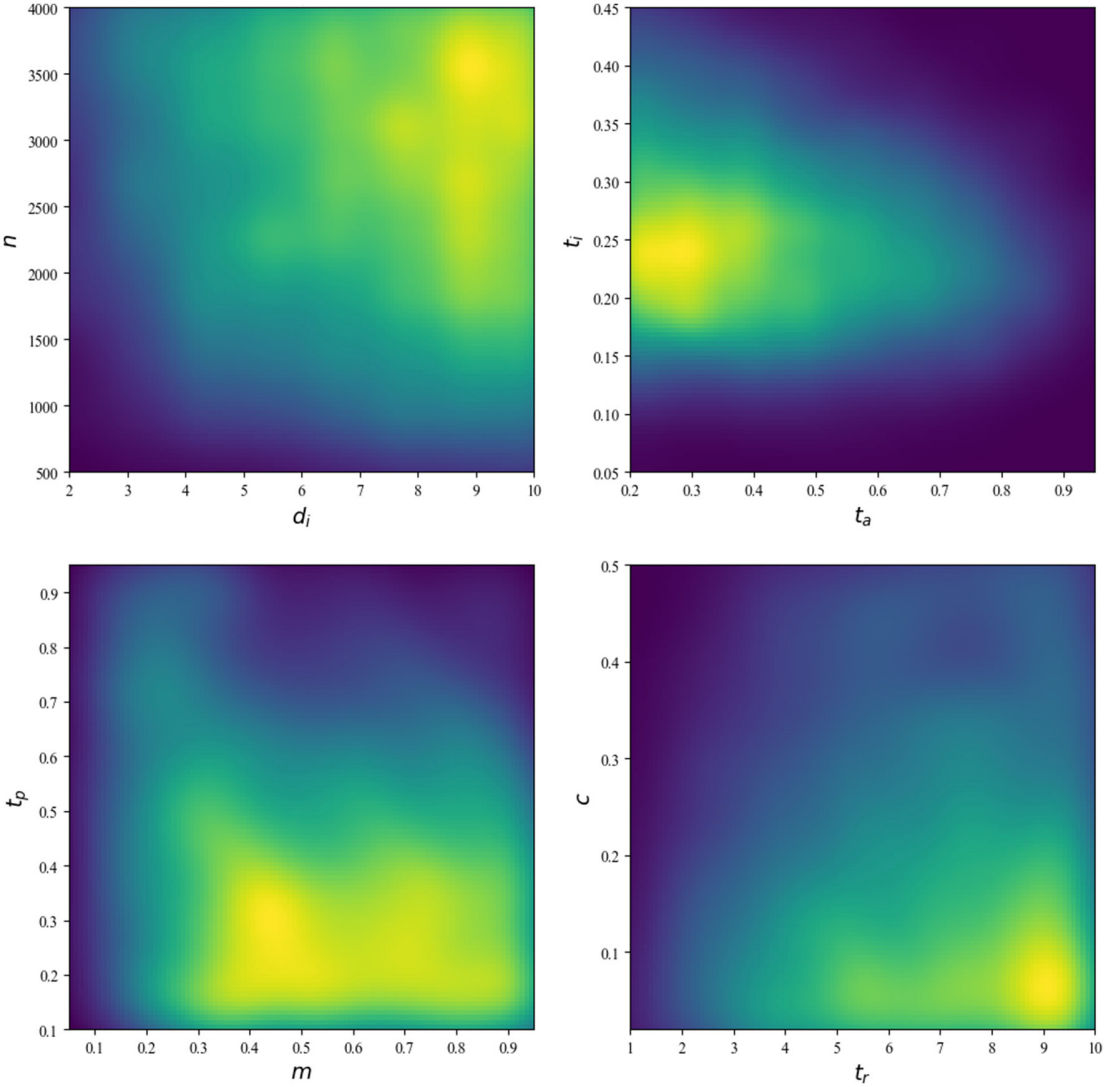
The number of simulations conducted for a configuration of parameters in *S*_*i*_. The lighter the heatmap, the more simulations there are at that location in parameter space. In contrast, a darker color describes a location in the parameter space where there are no simulations.

It is evident that there is a co-effect between the environmental parameters, which can be visualized in Figure 8. The gradient of the simulation in the top-left part of Figure 8 exhibits a pronounced clustering of elevated values in the top right quadrant, with a gradual decline in the lower left. This suggests that, within the confines of this experimental configuration, it is highly improbable to observe a simulation characterized by low population density and minimal infection duration. Analogously, the top-right subfigure illustrates that, for a given configuration of infection threshold (*t*_*c*_) and attendance threshold (*t*_*a*_), the probability of infection survival is higher when the majority of runs occur at a specific point.

## 5 Discussions and conclusions

This paper introduces a modified version of the El Farol Bar social dilemma with an added epidemiological dimension, where individuals must choose whether to engage in social activities during a disease outbreak. The simulation results indicate two key messages. The first is the importance of the rate of information available in the social system, which can be observed in the role of short-term memories and personal awareness regarding the infective status. This is not unexpected when the model is viewed as a decision-making context, and there is a long tradition of research exploring how an entity's capacity to gather and process information affects their actions. This aligns with Soros's conceptual framework, particularly the 'principle of fallibility,' which asserts that individuals' perceptions of reality are inherently flawed due to biases or inconsistencies (Soros, 1988, 2013). In the context of social systems, these imperfect views influence decision-making processes, shaping the outcomes of collective actions. Also, the effect can be explained by the “principle of reflexivity" (Soros, 1988, 2013), which plays a pivotal role by illustrating how individual beliefs—such as misconceptions about infection risks—not only influence personal behaviors but also modify the dynamics of the social and epidemiological systems as a whole. For example, if a majority of agents underestimate its contagiousness, their actions can exacerbate disease spread, thus reshaping the trajectory of the epidemic and the social structure itself (Beinhocker, 2013).

The second element is the need for taking explicitly into account social system structure, which in this case derives from agents' numerosity and dynamics. From a topological perspective, an agent can be situated in one of two states: present or absent from the event. Agents that are present are interconnected, at least potentially, while those that are absent are not. This situation undergoes change at each time step. These elements can change in accordance with both social and epidemic features, which in turn give rise to a diversification of the system structure and, consequently, an increase in the probability of an infection lasting. Also, the results indicate a positive effect between population size and infection surviving. Given than, by design, the model is a structured representation of a closed systems, this finding suggests that an efficient strategy could be involve sectoral management by dividing larger systems into smaller, manageable units. Such an approach would facilitate easier control and monitoring of these smaller systems.

Moreover, the role of memory in this model could provide important insights about decision-making in dynamic social-epidemiological systems. On one hand, the implication appears straightforward: agents who focus more on recent events, rather than relying on a longer history of past experiences, are better positioned to make adaptive decisions about whether to attend the bar. This tendency to prioritize the present over distant past events helps break the synchronization of attendance patterns, reducing crowd-induced infection risks and stabilizing system dynamics. However, this insight challenges conventional wisdom in data-driven decision-making. A big data expert might argue that having more data–such as an extended, detailed time series–should lead to better decisions. The model, however, reflects the cognitive constraints of the agents, who function with limited capacity for processing complex information. In scenarios where cognitive capacity is low (or where agents behave as if it is), focusing on the most recent information proves to be a more effective strategy than relying on an exhaustive analysis of historical trends. This finding underscores an important tradeoff: when resources for processing information are constrained, simplicity can be advantageous. It suggests that decision-making entities—whether individuals, organizations, or automated systems–may benefit from heuristics that prioritize recent data over large datasets in certain contexts.

The most negative effect on infection spread is linked to the infectiousness threshold. This is consistent with the expectation that when an infective specimen falls in the area where can be perceived longer than it can be widespread, it has less chances to survive. Furthermore, this underscores again the critical role of information. More informed agents, who are thus more aware of the risks, and so have an higher perception threshold, are more likely to make decisions that would spread the infection, thereby influencing the overall dynamics of disease spread.

The findings from this paper could have practical implications for real-world scenarios, particularly in public health management during epidemics. First, the importance of information flow and short-term memory highlights the need for timely and accurate communication strategies. Public health authorities could design targeted messaging campaigns that provide clear and actionable information to the public, enabling individuals to make informed decisions that reduce disease transmission. Second, insights into how agents' perceptions and crowd dynamics influence infection spread could inform the development of adaptive social distancing measures. For instance, policies that dynamically adjust venue capacity limits based on real-time occupancy or local infection rates could effectively prevent overcrowding and minimize transmission risks. Lastly, the role of personal awareness and memory in decision-making underscores the value of sustained public health campaigns. Educational initiatives that build long-term awareness of health risks and encourage proactive behaviors, such as wearing masks or staying home when symptomatic, can help maintain a higher level of community preparedness and resilience during future outbreaks.

A limitation of this study is the homogeneity of agents' behavior. Future research could explore how the system behaves when agents exhibit diverse behaviors. However, it was deemed unnecessary to include this aspect in the initial analysis. Intuitively, increasing the number of agents would likely result in behavioral heterogeneity being averaged out in a mean-field approximation, which guided the decision to use the current configuration. Nonetheless, this represents a limitation and a potential avenue for future research aimed at validating the results under more heterogeneous scenarios.

Also, the endogeneity of contagion risk perception deserves more exploration. In our model, agents' decisions are based on memory and expectations regarding social attendance but do not incorporate adaptive perceptions of contagion risk. However, in real-world scenarios, individuals often adjust their behavior dynamically based on perceived risk (Busby et al., 2016), which in turn alters the actual spread of the disease. If agents were to update their decision-making based on the prevalence of infections in their environment–either through direct experience or social influence—the model outcomes might change significantly. Higher perceived risk could lead to more cautious behaviors, effectively flattening the epidemic curve, while underestimation of risk could accelerate contagion.

More broadly, the main limitation of this study is that the analyses were conducted within the framework of a social dilemma, rather than on an empirically validated model. While the insights and results derived from this approach are valid, they should be viewed as preliminary suggestions for empirical researchers conducting field experiments. The findings provide a theoretical foundation that can guide future empirical work, but further validation through real-world data is necessary to confirm their applicability.

Future developments can take multiple directions. First, the time-series dynamics of the model could be studied to identify whether specific dynamic equilibria or limit cycles emerge, which could be highly relevant for public decision-makers. Second, an extension to the model could be make, introducing a policy-maker agent that could employ actions for epidemic containment. The results from the synthetic decision-makers' actions could be compared to real-world policy decisions made during the COVID-19 pandemic. Third, an analysis could be conducted to quantify the amount of information (in bits) required by each agent to make informed decisions in such contexts. Finally, a potential extension could explore long-term infections not tied to short-term events like a night out, but where the socio-epidemiological co-dynamics remain critical, such as in diseases like AIDS.

## Data Availability

The datasets presented in this study can be found in online repositories. The names of the repository/repositories and accession number(s) can be found at: https://anonymous.4open.science/r/epielfarol-D880/.
